# Cellulose-Based Flame-Retardant Materials: Mechanisms, Evaluation, and Design Strategies

**DOI:** 10.1021/acs.biomac.6c00936

**Published:** 2026-07-27

**Authors:** Jiansong Chen, Haishun Du, Xuejun Pan

**Affiliations:** † Department of Biological Systems Engineering, 5228 University of Wisconsin-Madison, Madison, Wisconsin 53726, United States; ‡ Department of Forestry, 3078 Michigan State University, East Lansing, Michigan 48824, United States

## Abstract

Cellulose-based materials are promising sustainable alternatives to petroleum-derived polymers because of their renewability, biodegradability, and structural versatility; however, their inherent flammability significantly limits their applications in fire-sensitive areas. This review presents a systematic overview of recent advances in flame-retardant cellulose-based materials, focusing on combustion fundamentals, fire-performance evaluation, and flame-retardant design strategies. The thermal decomposition behavior of cellulose is first discussed, emphasizing the competition between volatile generation and condensed-phase char formation that governs flammability. Common fire-performance evaluation methods, including thermogravimetric analysis, limiting oxygen index, vertical burning tests, and cone calorimetry, are then examined in the context of flame-retardant mechanisms. Subsequently, major preparation approaches—physical blending, surface coating, chemical modification, and grafting copolymerization—are comparatively reviewed. Representative phosphorus-, nitrogen-, boron-, silicon-, and metal-based systems are highlighted to illustrate condensed-phase, gas-phase, and synergistic flame-retardant effects. Finally, current challenges and future opportunities are discussed, focusing on durability, multifunctionality, mechanistic understanding, and scalable manufacturing. By integrating fundamental combustion principles with material design strategies, this review provides a framework for the rational development of sustainable, halogen-free, flame-retardant cellulose materials.

## Graphical abstract

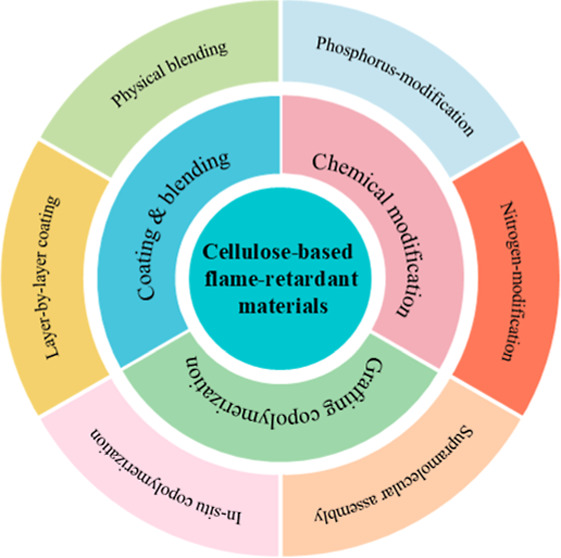

## Introduction

The increasing demand for sustainable and environmentally benign materials has stimulated growing interest in cellulose-based materials as alternatives to petroleum-derived polymers.^1,2^ As the most abundant natural biopolymer on Earth, cellulose possesses several attractive features, including renewability, biodegradability, wide availability, and versatile processability.^3–7^ When engineered into fibers, films, papers, foams, and aerogels, cellulose-based functional materials can exhibit excellent mechanical performance and functional diversity, enabling their use in textiles, packaging, building materials, thermal insulation, and other advanced applications.^8–10^ However, their inherent flammability remains a major limitation, particularly in scenarios where fire safety is a critical requirement.^11^

Upon heating, cellulose undergoes dehydration, depolymerization, and fragmentation, generating flammable volatiles that can sustain combustion.^12,13^ Although char is formed during thermal decomposition, the native char layer is generally fragile and porous and therefore cannot effectively prevent heat transfer, oxygen diffusion, or further release of combustible products.^14,15^ For this reason, improving the flame retardancy of cellulose-based materials has become an important topic in both fundamental research and practical applications.^16^ Traditional approaches often relied on halogen-containing flame retardants or high loadings of inorganic additives, but these systems are frequently associated with concerns such as toxicity, environmental persistence, additive migration, and deterioration of material performance.^17–19^ These drawbacks have driven the search for halogen-free, durable, and more sustainable flame-retardant strategies for cellulose-based systems.^20,21^

In recent years, substantial progress has been made through both chemical regulation and structural design. At the molecular level, cellulose has been modified by introducing phosphorus-, nitrogen-, or boron-containing functionalities or by constructing covalently bonded and grafted flame-retardant structures that promote catalytic dehydration and char formation.^22,23^ At the structural level, strategies such as physical blending, surface coating, and interfacial assembly have been widely explored to create effective barrier layers or synergistic flame-retardant architectures.^24,25^ In addition, porous cellulose materials such as foams and aerogels provide unique opportunities for flame-retardant design, since their hierarchical structures can simultaneously influence heat transfer, volatile release, and combustion behavior.^26,27^ As a result, many recent studies have moved beyond single-function fire protection and toward multifunctional systems that integrate flame retardancy with mechanical robustness, thermal insulation, or other desirable properties.^28,29^

This research trend is also reflected in the keyword co-occurrence network shown in Figure 1a, which summarizes the research directions and emerging themes of cellulose-based flame-retardant materials. High-frequency terms such as flame retardant, flame retardancy, thermal stability, cotton fabric, and cellulose occupy central positions in the network, indicating that fire performance and substrate-specific design remain the core topics in this field. At the same time, the appearance of more recent keywords related to durability, phytic acid, layer-by-layer, and biobased modification suggests a clear shift from conventional flame-retardant treatment toward multifunctional, sustainable, and structurally tunable cellulose systems.

**Figure 1:**
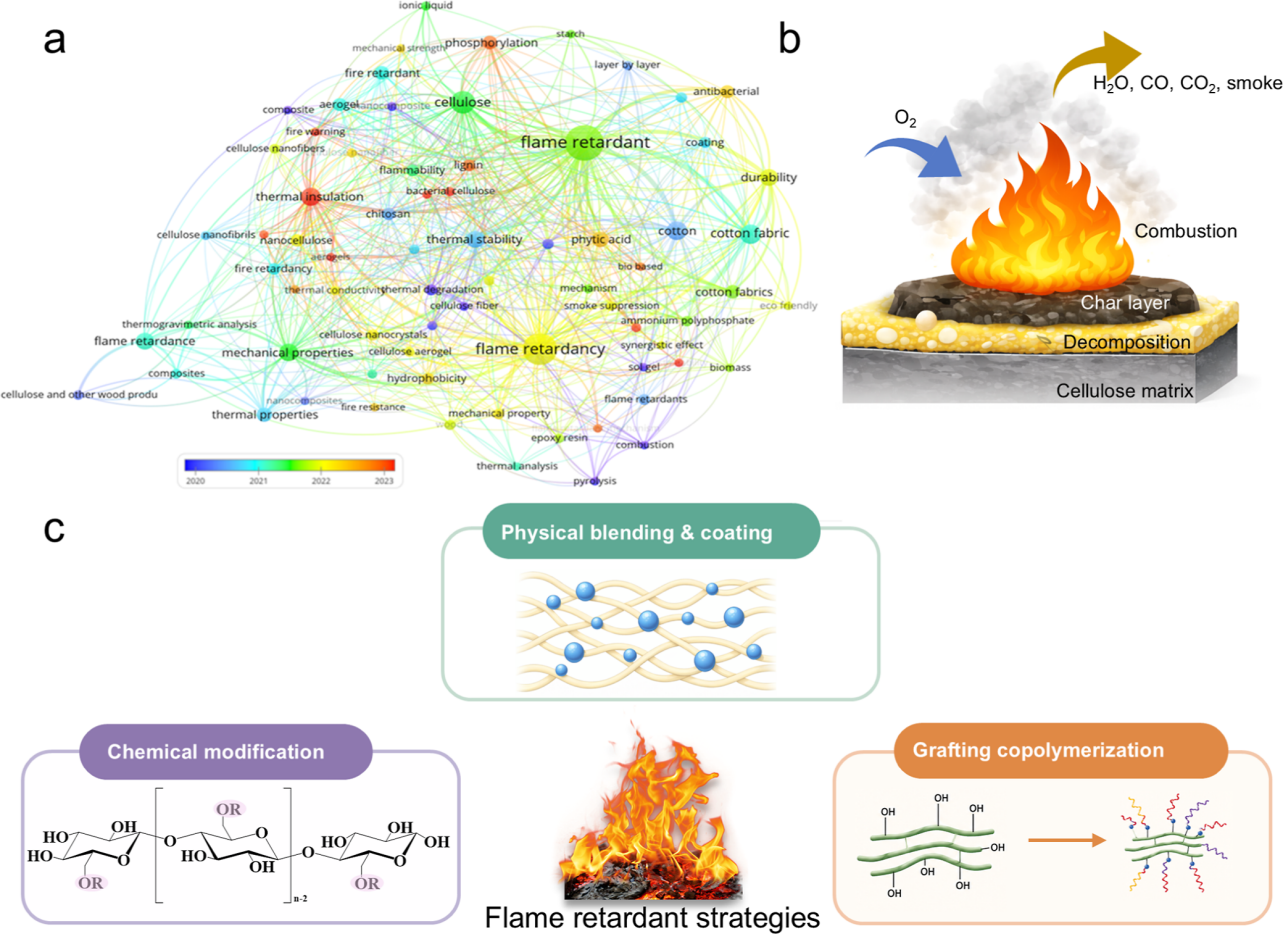
(a) Keyword co-occurrence network analysis based on Web of Science publications, illustrating the evolution and interconnections of major research themes related to cellulose flame retardancy, including flame-retardant performance, thermal stability, mechanical properties, and sustainable modification strategies. Node size reflects keyword frequency, and color indicates the average publication year. (b) Schematic illustration of the combustion behavior of cellulose materials, highlighting thermal decomposition of the cellulose matrix, formation of a char layer, and the release of gaseous products (H_2_O, CO, CO_2_, and smoke) during combustion. (c) Representative flame-retardant strategies for cellulose-based materials.

Despite these advances, the flame retardancy of cellulose-based materials is still governed by complex relationships among chemical composition, interfacial interactions, hierarchical structure, and macroscopic fire behavior.^30,31^ Limiting oxygen index (LOI), heat release rate (HRR), total heat release (THR), and smoke production depend not only on the type of flame retardant used,^32–35^ but also on how it is incorporated, distributed, and retained within the cellulose matrix. Although many studies have reported promising results, the literature remains somewhat fragmented, with different works focusing on specific additives, fabrication methods, or substrate forms. A clearer framework linking flame-retardant mechanisms, preparation strategies, and fire-performance outcomes is therefore needed.

Compared with previous reviews that primarily focus on specific flame-retardant additives, individual modification approaches, or particular cellulose substrates, this review places greater emphasis on establishing an integrated mechanism–evaluation–strategy framework for cellulose-based flame-retardant materials. Specifically, we correlate the intrinsic thermal decomposition behavior of cellulose, including volatile generation and char formation, with the corresponding condensed-phase and gas-phase flame-retardant mechanisms. We further discuss how these mechanisms are reflected in commonly used fire-performance evaluation methods and how they guide the rational design of physical blending, coating, chemical modification, and grafting copolymerization strategies. By linking fundamental degradation processes, flame-retardant mechanisms, performance assessment, and material design, the review provides a more comprehensive perspective on the interrelationships among chemical composition, structural engineering, and fire-performance outcomes. In addition to summarizing recent advances, we highlight emerging opportunities in sustainable, durable, and multifunctional cellulose-based systems, which represent an important direction for future development. In this review, we first summarize the thermal decomposition behavior of cellulose and the major flame-retardant mechanisms operating in the condensed and gas phases. We then introduce the commonly used methods for evaluating flame-retardant performance. After that, the main preparation strategies, including physical blending, coating, chemical modification, and grafting copolymerization, are discussed and compared. Finally, the major challenges and future perspectives in this field are outlined, with particular emphasis on the development of sustainable, durable, and multifunctional flame-retardant cellulose materials.

### Fundamentals of Flame Retardancy in Cellulose

Combustion of condensed-phase materials is generally sustained by the coupled transfer of heat, mass, and reactive species among the solid, gas, and flame zones.^36,37^ Upon external heating, a solid fuel first undergoes thermal decomposition to generate volatile combustible products, which diffuse into the gas phase, mix with oxygen, and ignite when the local temperature and composition become favorable.^38,39^ The heat released from gas-phase combustion is then fed back to the solid surface, further promoting pyrolysis and volatile evolution, thereby establishing a self-sustaining combustion cycle.^40,41^ Accordingly, flame-retardant strategies generally aim to interrupt this cycle either in the gas phase by quenching flame-propagating radicals or in the condensed phase by suppressing pyrolysis, reducing volatile release, and promoting the formation of a protective char layer.^42,43^

Cellulose is a linear polysaccharide composed of β-(1→4)-linked anhydroglucose units,^9,44^ and its thermal behavior is strongly governed by the abundance of hydroxyl groups, which promote extensive intra- and intermolecular hydrogen bonding.^45,46^ These hydrogen bonds tightly associate adjacent chains, thereby affecting chain packing, crystallinity, and moisture uptake.^47,48^ Figure 1b illustrates the fundamental combustion behavior of cellulose, including its dehydration, volatilization, char formation, and overall thermal degradation behavior.^49,50^ These processes lead to the cleavage of glycosidic bonds and the formation of low-molecular-weight oxygenated products, such as levoglucosan and other volatile species, which readily diffuse into the gas phase and sustain flaming combustion after ignition.^51,52^ Therefore, the high flammability of cellulose mainly originates from its strong tendency to generate combustible volatiles during thermal decomposition.^53^

At the same time, cellulose can also undergo dehydration and cross-linking reactions in the condensed phase, resulting in the formation of carbonaceous char.^54^ However, the char produced from pristine cellulose is usually discontinuous, porous, and mechanically fragile and thus provides only limited protection against heat transfer, oxygen diffusion, and further volatile release. As a result, the fire behavior of cellulose is essentially determined by the competition between two pathways during thermal decomposition: one favors the formation of flammable volatiles, while the other promotes char generation and stabilization. Effective flame-retardant strategies for cellulose, therefore, rely on shifting this balance toward the condensed phase by suppressing volatilization, enhancing catalytic dehydration, and improving the integrity and barrier function of the resulting char layer.

### Cellulose Flame-Retardant Property Evaluation

The flame-retardant performance of cellulose-based materials cannot be assessed by a single parameter because fire behavior is determined by multiple interconnected processes, including thermal decomposition, volatile release, ignition, flame spread, heat release, smoke generation, and char evolution. For this reason, the evaluation of flame retardancy should be considered as a multilevel process in which small-scale thermal analysis, flammability testing, and bench-scale fire characterization complement one another. Meaningful interpretation of these data also requires correlation with the intrinsic combustion pathways of cellulose discussed in Section 2, particularly the balance between volatile generation and condensed-phase carbonization.

At the first level, thermal analysis is commonly used to examine how flame-retardant treatments alter the decomposition pathways of cellulose. Thermogravimetric analysis (TGA) provides basic information on degradation onset, mass loss behavior, and residual char yield.^55^ In flame-retardant cellulose systems, an earlier onset of mass loss is not necessarily unfavorable; in many cases, it reflects the catalytic action of phosphorus-, boron-, or metal-containing species that promote dehydration and accelerate char formation at lower temperatures. Therefore, TGA results should not be interpreted solely in terms of thermal stability but rather in relation to whether the decomposition pathway is shifted from volatile production toward protective carbonization. Derivative thermogravimetry (DTG) further helps identify changes in degradation kinetics, such as the suppression of rapid depolymerization peaks and the development of broader decomposition features associated with gradual charring.^56^ However, thermal analysis alone cannot fully predict fire safety because a high char yield is beneficial only when the resulting char is sufficiently continuous and stable to act as an effective barrier. At the second level, flammability tests are used to evaluate ignition resistance and flame propagation under relatively simple conditions. The LOI is one of the most widely used indicators for cellulose-based materials, as it reflects the minimum oxygen concentration required to sustain combustion.^57^ In general, a higher LOI indicates lower flammability and improved resistance to sustained burning. For many flame-retardant cellulose materials, LOI enhancement is closely related to reduced volatile release and increased condensed phase protection. Vertical or horizontal burning tests, including UL-94 and related methods, provide additional information on self-extinguishing behavior, afterflame time, afterglow time, and dripping.^58^ These tests are useful for a rapid comparison of formulations, especially for textiles, foams, and lightweight composites. Nevertheless, because they are performed under relatively low and simplified heat exposure, they mainly reflect flammability ranking rather than the actual fire hazard.

Cone calorimetry is generally regarded as the most informative method for evaluating fire behavior under a realistic external heat flux.^59^ Compared with LOI or vertical burning tests, cone calorimetry provides a more complete picture of combustion intensity and fire growth. Among the measured parameters, the HRR, peak HRR (pHRR), THR, and time to ignition are particularly important for cellulose-based materials. A reduction in pHRR usually indicates suppression of rapid combustion and lower fire intensity, while a decrease in THR suggests a reduced overall contribution of the material to the fire load. Delayed ignition may reflect improved thermal shielding, lower heat feedback, or a reduced availability of flammable volatiles. In cellulose-based flame-retardant systems, these changes are often associated with enhanced char formation, barrier layer development, or reduced mass and heat transfer during burning.

Beyond these basic parameters, the shape of the HRR curve can also provide useful mechanistic information. A broader and lower HRR peak often suggests that combustion has been slowed by barrier effects or gradual release of degradation products. In contrast, secondary HRR peaks may indicate structural collapse, rupture of the protective char layer, or renewed exposure to unburned material. Similarly, smoke production, carbon monoxide release, and related toxic gas data are increasingly important in evaluating fire safety, particularly for porous cellulose materials such as foams and aerogels. Because these materials often burn rapidly and may release large amounts of volatile products, flame-retardant designs should ideally reduce not only heat release but also smoke and toxic gas evolution.

Finally, morphological and chemical characterization of the combustion residue is essential for linking fire performance to flame-retardant mechanisms. Techniques such as scanning electron microscopy (SEM), Fourier transform infrared spectroscopy, Raman spectroscopy, and X-ray photoelectron spectroscopy are frequently used to examine char continuity, compactness, graphitization degree, and elemental distribution after burning.^60^ These analyses help determine whether a flame-retardant system acts mainly through catalytic charring, formation of an inorganic protective layer, intumescence, or gas-phase inhibition. Therefore, a reliable evaluation of flame-retardant cellulose materials should combine thermal analysis, flammability testing, cone calorimetry, and postcombustion residue characterization. Only through such an integrated approach can the true fire performance of cellulose-based materials be understood and compared in a meaningful way.

### Strategies for Preparing Cellulose-Based Flame-Retardant Materials

To improve the flame retardancy of cellulose-based materials, it is necessary to consider the general principles that govern combustion suppression. In most cases, effective flame-retardant design aims to alter the thermal decomposition behavior of cellulose so as to reduce the generation of flammable volatiles, promote char formation, and construct protective barriers that limit heat transfer, oxygen diffusion, and mass transport during combustion.^61,62^ Depending on the flame-retardant system, additional effects may also contribute, such as endothermic heat absorption, release of noncombustible gases for dilution, or gas-phase radical quenching.^63,64^ On the basis of these general principles, a variety of preparation strategies have been developed for cellulose-based flame-retardant materials, among which physical blending, coating, chemical modification, and graft copolymerization are the most representative (Figure 1c). Although these approaches differ in their design principles and levels of interaction with the cellulose matrix, they all aim to regulate the thermal decomposition pathway of cellulose, suppress the release of flammable volatiles, and promote the formation of protective condensed phase structures during combustion. In general, physical blending and coating are relatively simple and versatile methods that rely on additive incorporation or surface protection, whereas chemical modification and graft copolymerization provide stronger interfacial or covalent integration of flame-retardant functionalities, leading to improved durability and design flexibility. A comparative summary of representative preparation methods, key flame-retardant characteristics, and typical performance outcomes is provided in Table 1. The following sections summarize these four major preparation strategies and discuss their respective mechanisms, advantages, and limitations in the development of flame-retardant cellulose materials.

**Table 1:** Summary of Representative Preparation Strategies, Mechanisms, and Flame-Retardant Characteristics of Cellulose-Based Flame-Retardant Materials

strategy	material	preparation method	key feature	ref
physical blending	microcrystalline cellulose (MCC) and nano-crystalline cellulose (NCC)	melt blending of polylactic acid (PLA) with cellulose particles and aluminum phytate	NCC/Al-phytate reduced pHRR from 390 to 240 kW m^–2^; nanoscale cellulose promoted cohesive char formation, while MCC showed a weaker effect	^95^
	cellulose fiber	resorcinol bis (diphenyl phosphate) RDP adsorption on cellulose fibers followed by melt blending with PLA	8 wt % coated cellulose-RDP enabled UL-94 V-0, self-extinguished within 2 s, reduced dripping, and improved tensile properties	^91^
	cotton	incorporation of MgAl-LDH into cellulose aerogels through NaOH/urea dissolution and freeze-drying	LDH reduced pHRR by 41–50%, reduced smoke production by 75–79%, and improved compression strength	^29^
	CNF	incorporation of sodium bicarbonate into CNF suspension followed by freeze-drying	burning velocity decreased from 5.84 to 0.20 cm s^–1^; thermal conductivity remained low at ∼28 mW m^–1^ K^–1^	^81^
surface coating	CNF	LbL assembly of chitosan/poly(vinylphosphonic acid)/chitosan/montmorillonite on CNF aerogels	five quadlayers enabled immediate self-extinguishing, no ignition under cone calorimetry at 35 kW m^–2^, and strong thermal shielding across 10 mm-thick aerogels	^85^
	cotton	dip-pad-dry coating of chitosan/phytic acid/Ba^2+^/phytic acid on cotton fabrics	biobased intumescent coating with only 5.2% weight gain; LOI increased from 16.2 to 22.0, and pHRR decreased from 333.1 to 129.1 W g^–1^	^90^
	cotton	LbL assembly of cationic chitosan and anionic phytic acid on cotton fabrics	fully renewable intumescent nanocoating; high-PA coating extinguished flame propagation and reduced pHRR and THR by 60% and 76%, respectively	^82^
	cotton	LbL assembly of cationic starch and poly (phosphoric acid) on cotton fabrics	only two bilayers gave self-extinguishing behavior with <5 wt % coating add-on; treated cotton showed up to 40% reduction in total heat release	^92^
chemical modification	CNF	phosphorylation of CNF with P_2_O_5_ by ball milling, followed by ionic bonding with melamine	P/N-containing CNF flame retardant improved bamboo paper; 30 wt % loading gave LOI of 30% and reduced pHRR by 62.8%, with self-extinguishing behavior and enhanced wet/dry tensile strength	^97^
	cotton	grafting amino acid–based P–N flame retardants onto cotton via P–O–C and C–O–C covalent bonds	formaldehyde-free treatment; 30% FR-L increased LOI to 42.1%, and LOI remained 27.1% after 40 laundering cycles, showing good durability	^99^
	wood flour	boric acid modification of lignocellulose through complexation/esterification before incorporation into polyamide 6	boric acid improved lignocellulose thermal stability and protected fibers during polyamide 6 processing, but negatively affected composite mechanical strength	^108^
	wood pulp/bamboo pulp/low lignin-containing bamboo pulp and bamboo powder	boric acid improved lignocellulose thermal stability and protected fibers during polyamide 6 processing, but negatively affected composite mechanical strength	phosphorus groups and lignin exhibited synergistic flame retardancy: pHRR and THR decreased by 87.3% and 86.6%, respectively, while the film retained high tensile strength and flexibility	^98^
grafting copolymerization	cellulosic pine needles	microwave-assisted grafting of butyl acrylate onto cellulose	grafting improved the moisture resistance, chemical resistance, and thermal stability of cellulose, showing the potential of graft copolymerization for durable functional cellulose materials	^100^
	bleached cotton fabrics	plasma-induced graft-polymerization of phosphorus/nitrogen-containing acrylate monomers on cotton	phosphoramidate monomers showed higher flame-retardant efficiency; diethyl(acryloyloxyethyl)phosphoramidate and cryloyloxy-1,3-bis(diethylphosphoramidate) propane increased LOI to 28.5 and 29.5, respectively, due to P–N synergistic effects	^105^

### Physical Blending of Flame Retardant into Cellulose Materials

Physical blending is a straightforward method to prepare flame-retardant cellulose materials by directly blending flame retardants into the cellulose matrix without altering its chemical structure.^29,65–67^ This approach offers advantages in simplicity, scalability, and compatibility with existing processing techniques, such as solution casting, freeze-drying, and melt processing.^68,69^ A wide range of flame retardants, including phosphorus-based compounds,^70^ nitrogen-containing additives,^71^ inorganic fillers (e.g., metal hydroxides and layered silicates),^72^ and intumescent systems, have been successfully introduced into cellulose substrates through physical mixing.^73^ From a mechanistic perspective, physically blended flame retardants typically function through condensed-phase char formation, gas-phase dilution, or a combination of both.^74^ For instance, phosphorus-based additives can promote dehydration and facilitate the formation of a protective char layer, while nitrogen-containing compounds may release nonflammable gases that dilute combustible volatiles.^75^ Inorganic fillers, on the other hand, often act as thermal barriers and heat sinks, reducing heat transfer and delaying thermal degradation.^76^ The effectiveness of physical blending is, therefore, highly dependent on the dispersion, compatibility, and interfacial interactions between the additives and the cellulose matrix.^77^

A representative example of physical blending is the incorporation of inorganic flame retardants into cellulose nanofiber (CNF) aerogels, as illustrated in Figure 2a. In the study by Cheng et al.,^78^ zinc borate was directly blended with cellulose nanofibers, followed by freeze-drying to form composite aerogels. The SEM image shows that the pristine cellulose aerogel exhibits a lamellar macroporous structure, which contributes to its low thermal conductivity, but also leads to high flammability. Upon introduction of zinc borate, the flame retardancy was significantly improved, as evidenced by a reduction in pHRR and THR, along with increased char residue. This enhancement is mainly attributed to the synergistic effects of zinc borate, including the formation of a protective B_2_O_3_ layer that acts as a physical barrier, the release of water and Zn-containing species that dilute combustible gases, and the promotion of char formation in the condensed phase. Importantly, this physical blending strategy achieved effective flame retardancy at relatively low additive loadings (e.g., 2 wt %) without significantly compromising the porous structure or thermal insulation performance of the aerogel, highlighting its practical potential.

**Figure 2:**
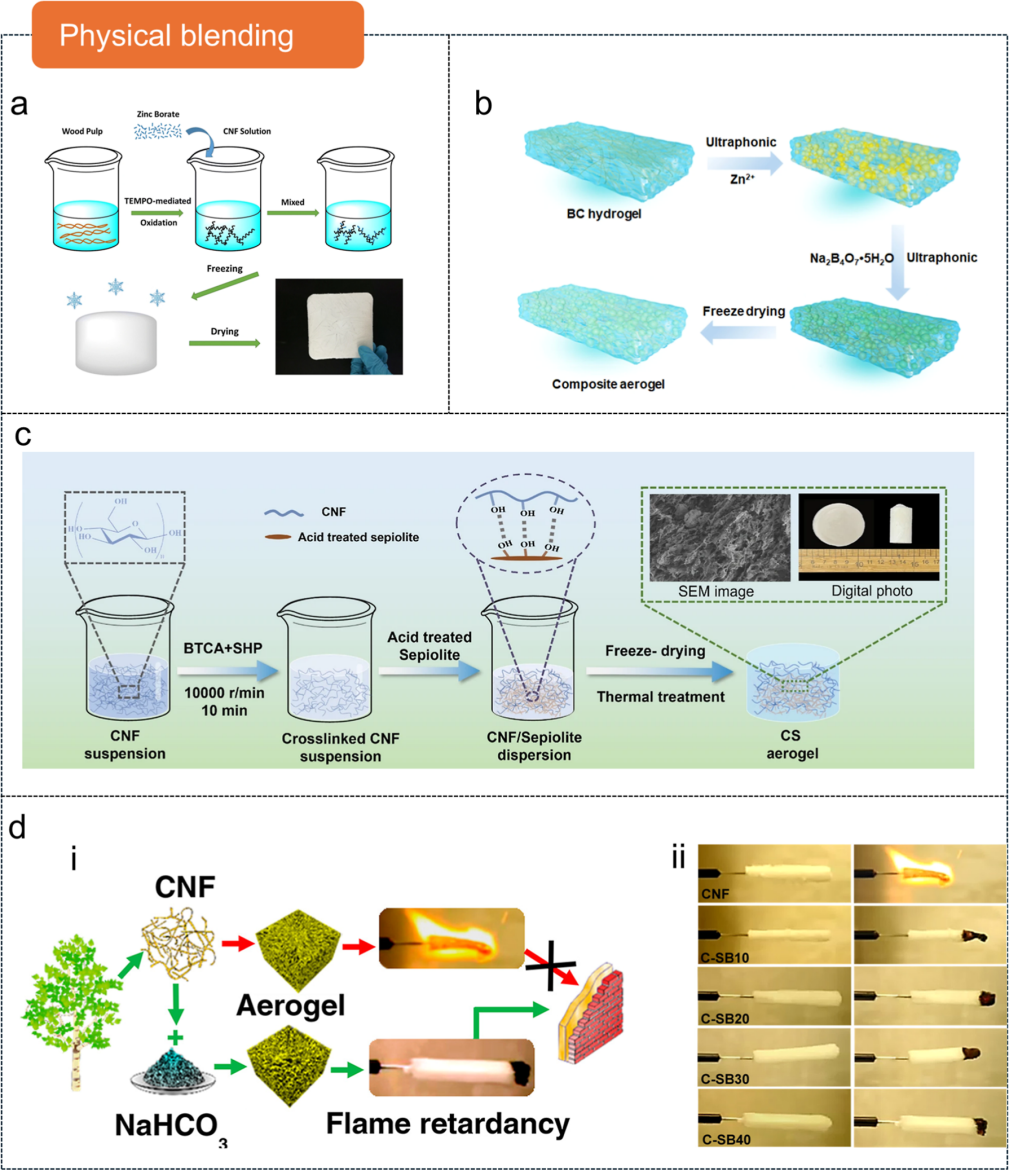
Representative physical blending strategies for preparing flame-retardant cellulose-based materials. (a) CNF/zinc borate aerogel. Adapted with permission.^78^ Copyright 2020, Springer. (b) Bacterial cellulose/zinc borate composite aerogel. Adapted with permission.^79^ Copyright 2023, Springer. (c) CNF/sepiolite aerogel composite. Adapted with permission.^80^ Copyright 2025, Springer. (d) Sodium bicarbonate-filled cellulose nanofibril aerogel. Adapted with permission.^81^ Copyright 2018, American Chemical Society under CC BY 4.0.

Similarly, a related but structurally distinct strategy was reported for bacterial cellulose aerogels by Wang et al.,^79^ as illustrated in Figure 2b, where zinc borate was introduced through an ultrasound-assisted in situ deposition process rather than simple premixing. In this system, the in situ formed zinc borate particles were uniformly deposited within the bacterial cellulose network, leading to the separation of individual fibrils from fiber bundles while preserving the overall porous framework. This more integrated distribution not only improved flame retardancy but also enhanced thermal stability and heat-insulation behavior. Quantitatively, the composite aerogel exhibited an extremely low heat release capacity of only 8 J g^–1^ K^–1^, together with dramatic reductions in pHRR and THR. The flame-retardant effect was attributed to the endothermic dehydration of zinc borate, which lowered the surface temperature by releasing bound water, as well as the in situ generation of ZnO and B_2_O_3_ that formed a continuous protective layer to retard heat transfer and isolate combustible cellulose fibrils.

Recent studies have explored the incorporation of multifunctional or nanostructured flame-retardant systems to achieve synergistic effects, as illustrated in Figure 2C. In the study by Wu et al.,^80^ hybrid additives were introduced into the cellulose matrix, forming a more integrated network structure that enhanced both flame retardancy and structural stability. Compared with conventional intumescent systems, these hybrid systems combine multiple functionalities such as catalytic charring, barrier formation, and gas-phase dilution. Upon thermal exposure, the additives promote the formation of a more compact and continuous char layer while simultaneously reinforcing the structural integrity of the residue, thereby improving its resistance to heat and mass transfer. In addition, the nanoscale dispersion of these components facilitates stronger interfacial interactions with the cellulose matrix, leading to a more efficient flame-retardant performance at relatively low loadings. This example highlights the emerging trend of designing synergistic and hierarchical flame-retardant systems through physical blending, with the aim of overcoming the limitations of single-component additives.

Environmentally benign gas-releasing additives have also been employed to improve the fire safety of cellulose aerogels, as illustrated in Figure 2d. In this case,^81^ sodium bicarbonate was incorporated into cellulose nanofibril aerogels through suspension mixing followed by freeze-drying, providing a simple and green route to flame-retardant materials. Unlike inorganic barrier-forming fillers such as zinc borate or sepiolite, sodium bicarbonate mainly acts through endothermic decomposition and gas-phase dilution. Upon heating, it absorbs heat and decomposes to release carbon dioxide and water, which dilutes the combustible atmosphere and suppresses flame propagation. As a result, the burning velocity of the aerogel decreased dramatically, and at sufficiently high loading, the material exhibited self-extinguishing or flameless pyrolysis behavior. Notably, this strategy improved flame retardancy without significantly sacrificing thermal insulation, since the thermal conductivity remained nearly unchanged at around 28 mW m^–1^ K^–1^. This example demonstrates that physical blending is not limited to solid barrier-type additives, but can also exploit thermally decomposable, eco-friendly additives to achieve effective flame retardancy in cellulose-based aerogels.

From a structure–property perspective, the flame-retardant performance of physically blended systems is governed primarily by the dispersion state, interfacial compatibility, and barrier-forming capability of the additives. Uniformly dispersed inorganic nanosheets, such as layered double hydroxide (LDHs), MXene, and montmorillonite can create tortuous pathways that suppress heat and mass transfer, whereas phosphorus-containing additives promote condensed-phase char formation. For example, Luo et al.^29^ incorporated MgAl-LDH into cellulose aerogels, where the uniformly dispersed LDH nanosheets enhanced the barrier effect and reduced the pHRR by 41–50% while also suppressing smoke production. This indicates that the effectiveness of physical blending depends not only on additive loading but also on additive dispersion, decomposition behavior, and interaction with the cellulose matrix during combustion.

Overall, physical blending is one of the most straightforward and industrially scalable approaches for producing flame-retardant cellulose materials, because it does not require chemical reactions or complex processing. In addition, a wide variety of flame retardants can be incorporated into this strategy. However, the flame-retardant performance strongly depends on the dispersion and compatibility of additives within the cellulose matrix. Additive migration, leaching, and deterioration of mechanical properties are also common challenges, particularly at high flame-retardant loading.

### Coating Cellulose Materials with a Flame-Retardant and Protective Layer

Coating is another effective strategy for fabricating flame-retardant cellulose-based materials by depositing a functional protective layer on the surface of the cellulose substrate rather than incorporating additives throughout the bulk matrix. Compared with physical blending, this approach is particularly attractive because it can impart flame retardancy while largely preserving the intrinsic lightweight structure, porosity, flexibility, or mechanical integrity of the original cellulose material. Depending on the design, the coating layer may be formed by dip-coating,^82^ spray-coating,^83^ sol–gel deposition,^84^ layer-by-layer assembly,^85^ or in situ surface mineralization^86,87^ and can consist of inorganic particles, polyelectrolytes, biobased macromolecules, intumescent components, or hybrid organic–inorganic systems.^88^ The flame-retardant behavior of coated cellulose materials is closely related to the architecture and integrity of the coating layer. Dense and continuous coatings provide more effective thermal shielding and oxygen barriers than discontinuous coatings.

From a mechanistic standpoint, coating-based flame retardancy mainly relies on the creation of an interfacial barrier between cellulose and an external heat source. Upon thermal exposure, the coating can suppress heat transfer, reduce oxygen diffusion, and hinder the release of volatile combustible products. In many systems, the coating further promotes the formation of a compact or intumescent char layer, thereby reinforcing the condensed-phase protection. If the coating contains gas-releasing or radical-quenching species, then additional gas-phase inhibition may also occur. The flame-retardant performance of coating systems depends strongly on the coating uniformity, thickness, adhesion to the cellulose surface, and structural stability during combustion.^89^

A representative example of the coating strategy is the deposition of a biobased intumescent system on cotton fabrics, as illustrated in Figure 3a. In the work by Zhang et al.,^90^ chitosan and phytic acid were assembled on the cellulose surface through a dip-pad-dry process, while barium ions were further introduced as a synergistic component to construct a CH/PA/Ba/PA coating with enhanced flame-retardant efficiency. Unlike bulk blending, this surface-confined coating concentrated the active flame-retardant components at the fiber interface, where ignition and thermal decomposition first occur. As a result, the coated fabric exhibited markedly reduced heat release, with the pHRR and THR decreasing from 333.1 to 129.1 W g^–1^ and from 13.1 to 5.4 kJ g^–1^, respectively, while the char residue increased substantially. The improved performance was attributed to the synergistic action of the biobased intumescent coating and metal ions: phytic acid promoted dehydration and char formation, chitosan served as both a carbon source and gas source, and barium ions catalyzed cross-linking and enhanced the integrity of the intumescent char layer. Importantly, this approach achieved effective flame retardancy with a relatively low add-on (∼5.2 wt %), suggesting that coating can provide efficient surface protection without severely compromising the softness of the original cotton substrate.

**Figure 3:**
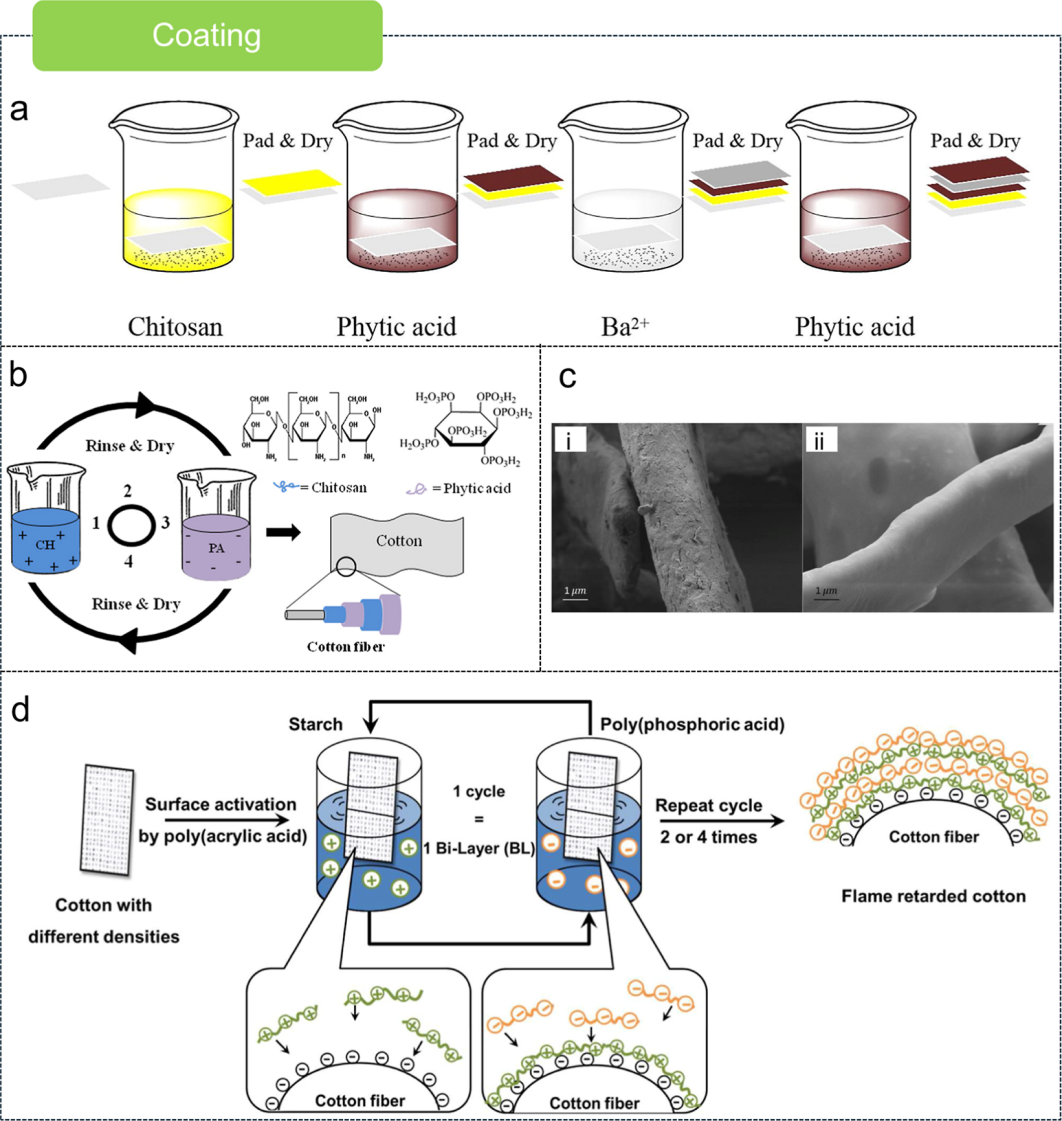
Representative coating and chemical-modification strategies for flame-retardant cellulose-based materials. (a) Chitosan/phytic acid/barium ion intumescent coating on cotton. Adapted with permission.^90^ Copyright 2019, Elsevier. (b) Layer-by-layer assembled chitosan/phytic acid nanocoating. Adapted with permission.^82^ Copyright 2012, American Chemical Society. (c) SEM images of cellulose fibers before and after flame-retardant coating. Adapted with permission.^91^ Copyright 2017, Elsevier. (d) Representative routes for intrinsic flame-retardant chemical modification of cellulose. Adapted with permission.^92^ Copyright 2015, American Chemical Society.

Another representative coating strategy is the construction of an intumescent multilayer nanocoating by layer-by-layer assembly, as illustrated in a study by Laufer et al.^82^ (Figure 3b). In this system, oppositely charged chitosan and phytic acid were alternately deposited on cotton fibers to form an ultrathin, conformal coating that uniformly covered the fiber surface. Unlike conventional bulk coatings, this nanoscale architecture enabled precise control over the coating thickness and phosphorus content, which strongly influenced flame-retardant performance. Fabrics coated with CH/PA multilayers showed markedly reduced flammability, and the coating prepared at lower pH with higher phytic acid content exhibited the best performance, reducing the pHRR and THR by about 60% and 76%, respectively, while largely preserving the fabric after vertical burning. This improvement was mainly attributed to the typical intumescent action of the CH/PA system: phytic acid acted as an acid source to catalyze dehydration of cellulose, whereas chitosan served as both a carbon source and a blowing agent, promoting the formation of a swollen multicellular char layer that insulated the underlying fibers from heat and oxygen. This example highlights the unique advantage of coating-based strategies in creating highly efficient flame-retardant protection through thin, conformal, and compositionally tunable surface layers.

A different surface-coating strategy was reported by Guo et al.,^91^ as illustrated in Figure 3c. The SEM images show the morphological change of cellulose fibers before (i) and after (ii) phosphate adsorption. In this system, cellulose fibers were coated with resorcinol bis­(diphenyl phosphate) (RDP), and the SEM images clearly reveal that the initially rough fiber surface became much smoother after coating, indicating that RDP uniformly wetted and covered the cellulose surface. This interfacial coating played a dual role: it immobilized the liquid phosphorus-containing flame retardant on the cellulose surface through hydrogen-bonding interactions and improved compatibility with the polymer matrix. As a result, the resulting PLA composite containing RDP-coated cellulose fibers showed markedly improved flame retardancy, including self-extinguishing behavior within 2 s and a clear increase in LOI to 28.0, while also maintaining or even enhancing the mechanical performance. Mechanistically, the coating promoted dehydration and char formation during burning while reducing dripping and heat release, demonstrating that surface coating of cellulose can be an effective way to integrate flame-retardant efficiency with interfacial reinforcement.

A further coating-based strategy is represented by layer-by-layer (LbL) assembly of biobased or hybrid intumescent nanocoatings, as illustrated in Figure 3d. In the study by Carosio et al.,^92^ cationic starch and poly­(phosphoric acid) were alternately deposited on cotton fabrics to form a thin, homogeneous LbL coating, and even only 2 bilayers were sufficient to impart self-extinguishing behavior with less than 5 wt % add-on, nearly doubling the thermally stable residue and reducing the THR by up to about 40%. A closely related approach was later extended to cellulose nanofibril aerogels,^85^ where chitosan, poly­(vinylphosphonic acid), and montmorillonite were assembled into a hybrid quadlayer coating that combined the essential intumescent components with inorganic nanoplatelets. Although the substrates differed, both studies relied on the same fundamental design principle: the surface-deposited multilayers acted as highly efficient interfacial flame-retardant architectures, in which the phosphorus-containing component promoted dehydration, the polysaccharide served as a carbon source, and the inorganic phase or multilayer structure reinforced the integrity of the resulting char barrier. As a consequence, these coatings markedly suppressed volatile release and heat transfer during combustion, showing that LbL assembly is a particularly powerful coating strategy for cellulose materials because it enables nanoscale control of composition, high flame-retardant efficiency at relatively low addition, and effective adaptation to both dense fabrics and highly porous aerogels.

Surface coating can effectively improve flame retardancy while minimizing changes to the bulk properties of the cellulose substrates. Moreover, highly efficient barrier structures can be generated at relatively low add-on levels. Nevertheless, coating durability remains a major concern, as coating layers may be subject to abrasion, washing-induced removal, or environmental degradation during long-term service.

### Chemical Modification of Cellulose to Improve Flame Retardancy

Chemical modification and grafting copolymerization are both covalent strategies for improving the flame retardancy of cellulose-based materials, and they may overlap in some of the reported systems. In this review, they are distinguished according to the dominant reaction pathway and the resulting molecular architecture. Chemical modification refers primarily to reactions that directly introduce flame-retardant functional groups or small molecular moieties onto cellulose, usually through the hydroxyl groups of the anhydroglucose units. Typical examples include phosphorylation, esterification, etherification, boronate esterification, and silylation. These reactions mainly change the chemical composition of cellulose and introduce phosphorus, nitrogen, boron, silicon, or other flame-retardant elements that can promote dehydration, suppress volatile formation, enhance char formation, or participate in gas-phase radical quenching. Chemical modification represents one of the most effective and durable strategies for imparting flame retardancy to cellulose-based materials,^93^ as it enables the covalent incorporation of functional groups into the cellulose backbone, thereby minimizing additive migration and enhancing long-term stability.^94^ Among various approaches, nitrogen- and/or phosphorus-based modifications have been demonstrated to be particularly effective, as they can fundamentally regulate the thermal decomposition pathway of cellulose by shifting the balance from volatile formation toward condensed-phase char generation.^66^ Representative strategies include covalent grafting of phosphorus- or nitrogen-containing molecules, homogeneous phosphorylation and esterification, derivative-level design of phosphorus-containing cellulose esters, and mechanochemical phosphorylation of nanocellulose.^95,96^

A representative example of this strategy is the mechanochemical phosphorylation of cellulose nanofibrils followed by the incorporation of a nitrogen source such as melamine to construct a P–N synergistic system^97^ (Figure 4a). In this case, phosphate groups are covalently introduced onto cellulose via ball milling with phosphorus pentoxide, while melamine is subsequently associated through ionic interactions, enabling uniform distribution of flame-retardant functionalities at the nanoscale. The modified material exhibits significantly improved fire performance, with the LOI increasing to over 30% and the pHRR reduced by more than 60% compared to untreated cellulose. Mechanistically, phosphorus promotes dehydration and char formation in the condensed phase, whereas nitrogen releases inert gases that dilute combustible volatiles, resulting in a combined condensed-phase and gas-phase flame-retardant effect. This example demonstrates that integrating covalent *P*-functionalization with N-based synergists is an effective route to achieve both enhanced flame retardancy and structural reinforcement in cellulose systems.

**Figure 4:**
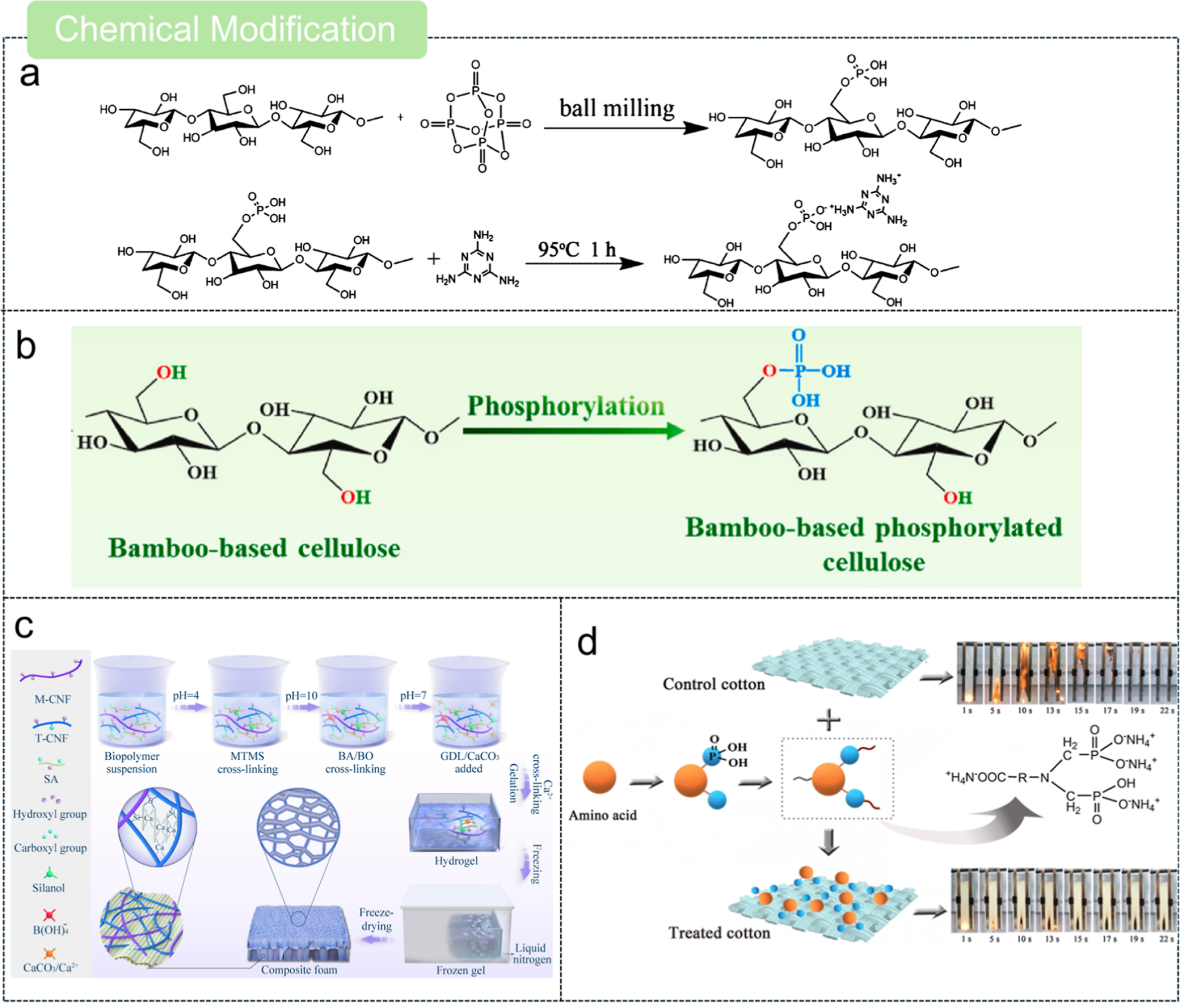
Representative chemical-modification routes for flame-retardant cellulose-based materials. (a) Mechanochemical phosphorylation of cellulose nanofibrils, followed by melamine incorporation to construct a phosphorus–nitrogen synergistic flame-retardant system. Adapted with permission.^97^ Copyright 2020, American Chemical Society. (b) Molecular grafting of amino-acid-derived phosphorus–nitrogen flame retardants onto cellulose through stable P–O–C linkages. Adapted with permission.^98^ Copyright 2021, Elsevier. (c) Borate-based chemical modification of cellulose composite foams through boric acid/borate incorporation, forming borate-related covalent structures that enhance condensed-phase protection. Adapted with permission.^27^ Copyright 2021, Elsevier. (d) Homogeneous phosphorylation/esterification of cellulose chains for the uniform introduction of phosphorus-containing groups and controlled molecular design of flame-retardant functionality. Adapted with permission.^99^ Copyright 2019, Springer.

Building on this nanoscale P–N synergistic strategy, similar concepts have also been extended to molecular-level grafting systems, where flame-retardant functionalities are covalently anchored onto the cellulose backbone. For instance, as illustrated in Figure 4b, Zhang et al.^98^ reported the design of amino-acid-derived phosphorus–nitrogen flame retardants that were chemically grafted onto cellulose through stable P–O–C linkages. This system exhibited markedly enhanced flame retardancy, with substantial increases in LOI and improved char formation ability. The improved performance was attributed to the combined effect of phosphorus-induced dehydration and nitrogen-enhanced char stabilization, which together promoted the formation of a compact and continuous protective layer during combustion. Compared with nanofibril-based systems, this approach highlights the effectiveness of molecular-level functionalization in achieving durable and tunable flame-retardant performance in cellulose materials.

In addition to phosphorus- and nitrogen-based systems, boron-containing chemical modifications have also been explored as an effective route to improve the fire safety of cellulose-based materials, as illustrated in Figure 4c. Boric acid/borate was introduced into cellulose-based composite foams together with sodium alginate and silane cross-linking. Spectroscopic analysis confirmed the formation of borate-related covalent structures, including monochelate and bis-chelate linkages between borate species and cellulose chains. Such chemical interactions altered the degradation behavior of the cellulose framework and promoted condensed-phase protection during combustion. As a result, the resulting foams showed self-extinguishing behavior and a markedly increased LOI of up to 39.5%, together with improved char formation and reduced fire growth. Mechanistically, the borate species contributed to the formation of a glassy protective layer upon heating while also favoring carbonization and suppressing cellulose depolymerization. This example suggests that beyond the widely studied P/N-based routes, covalent borate chemistry can also serve as a useful chemical modification strategy for regulating the combustion behavior of cellulose materials.

Extending this molecular-level grafting strategy, further efforts have focused on more uniform and controllable functionalization along the cellulose backbone, as illustrated in Figure 4d. In this approach,^99^ phosphorus-containing groups are introduced via homogeneous phosphorylation or esterification, enabling precise regulation of the degree of substitution and a more even distribution of flame-retardant functionalities within the cellulose chains. Compared with surface grafting, this strategy leads to more consistent thermal behavior and enhanced char formation capability. The improved performance is attributed to the uniform generation of acidic species during thermal decomposition, which effectively catalyze dehydration reactions and suppress the release of flammable volatiles. As a result, the modified cellulose exhibits higher char yield and improved flame retardancy, highlighting the importance of controlled molecular design in optimizing structure–property relationships in P-based flame-retardant systems.

Chemical modification provides a more durable flame-retardant effect because the flame-retardant functionalities are covalently attached to cellulose chains. This approach can reduce additive migration and improve the long-term stability. However, the synthesis procedures are often more complex and may require additional reagents, solvents, or catalysts. Excessive chemical modification may also affect the intrinsic crystallinity and mechanical performance of the cellulose.

### Grafting Copolymerization for Durable Flame-Retardant Cellulose Materials

Unlike chemical modification, which typically introduces flame-retardant groups through direct reactions with cellulose hydroxyl groups, graft copolymerization involves the covalent growth of polymer chains from or onto the cellulose backbone, with these grafted chains imparting flame-retardant functionality.^100^ As a result, this strategy not only preserves the durability associated with covalent attachment but also provides greater flexibility in molecular design by allowing control over the composition, length, and density of grafted side chains.^101^ Because of this structural versatility, graft copolymerization is particularly attractive for the development of multifunctional cellulose materials with durable flame retardancy.^102,103^

A representative example of grafting-based flame-retardant design is shown in Figure 5a,^104^ where a boron–nitrogen synergistic finishing system was constructed on cotton fabric using boric acid and the nitrogen-containing triazine derivative Tri-HTAC. In this system, Tri-HTAC, which contains multiple reactive hydroxylated side groups and a nitrogen-rich triazine ring, was able to form covalent linkages with cellulose and simultaneously interact with boric acid, generating a cross-linked surface structure on the cotton fibers. As a result, the treated fabric exhibited a clear improvement in flame retardancy, with the LOI increasing from 17.5 for untreated cotton to above 27.5, while TGA further showed a much higher char residue at 600 °C. Mechanistically, the boron-containing component promoted early dehydration and glassy char formation, whereas the nitrogen-containing triazine structure contributed to synergistic condensed-phase protection, together suppressing cellulose decomposition and enhancing carbonization. This example illustrates that grafting reactive flame-retardant species onto cellulose can provide a durable and efficient route to improving fire resistance through cooperative boron–nitrogen effects.

**Figure 5:**
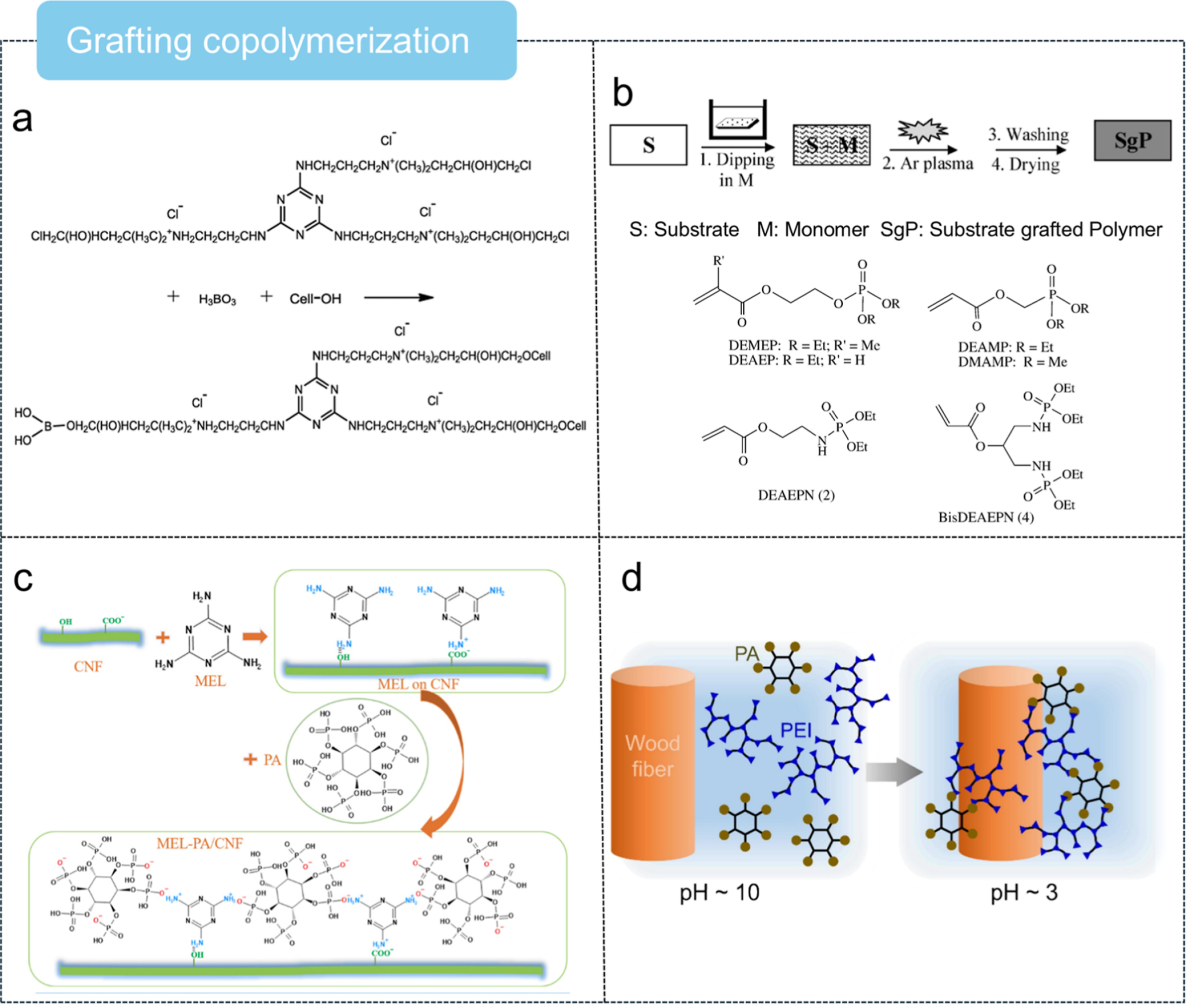
Grafting copolymerization-related strategies for flame-retardant cellulose-based materials. (a) Boron–nitrogen synergistic grafting on cotton. Adapted with permission.^104^ Copyright 2013, Elsevier. (b) Plasma-induced graft polymerization of phosphorus-containing monomers. Adapted with permission.^105^ Copyright 2006, Elsevier. (c) Melamine–phytic acid interfacial assembly on CNF aerogels. Adapted with permission.^106^ Copyright 2022, Wiley-VCH. (d) Phytic acid/polyethylenimine supramolecular assembly on cellulosic microfibers. Adapted with permission.^107^ Copyright 2024, Elsevier.

Another representative example of graft copolymerization is shown in Figure 5b by Tsafack et al.,^105^ in which plasma-induced graft polymerization was used to immobilize phosphorus-containing acrylate monomers on cotton surfaces through a covalently fixed polymer layer. In this strategy, reactive acrylate phosphate, phosphonate, and phosphoramidate monomers were first impregnated into the fabric and then simultaneously grafted and polymerized under argon plasma, producing a thin phosphorus-rich coating while preserving the breathability of the textile. Compared with untreated cotton, the modified fabrics showed greatly improved flame retardancy, with the LOI increasing from 19 to as high as 29.5 for the phosphoramidate-based system. Notably, monomers containing both phosphorus and nitrogen exhibited higher efficiency than phosphate- or phosphonate-only analogues at similar phosphorus contents, indicating a clear P–N synergistic effect. Mechanistically, the grafted phosphorus-containing polymer decomposed at relatively low temperature to generate acidic species that promoted phosphorylation of cellulose hydroxyl groups, suppressed levoglucosan formation, and shifted pyrolysis toward char formation. This example highlights the unique advantage of graft copolymerization in creating durable, surface-confined flame-retardant architectures with tunable monomer chemistry and enhanced washing resistance.

A further example, shown in Figure 5c, studied by Ren et al.,^106^ demonstrates that grafting-related flame-retardant design can also be achieved through supramolecular assembly on cellulose nanofibril aerogels, thereby extending the concept of surface-confined functionalization beyond conventional covalent polymer grafting. In this work, melamine and phytic acid were assembled in situ onto the cell walls of CNF aerogels to form rod-like MEL–PA nanostructures uniformly distributed throughout the porous network. Although the interactions were primarily based on hydrogen bonding and ionic association rather than classical chain-growth polymerization, the resulting architecture functioned similarly to a grafted interfacial flame-retardant layer by firmly anchoring nitrogen- and phosphorus-rich species onto the cellulose framework. As a result, the modified aerogel exhibited markedly improved flame retardancy, with the LOI increasing from 19.5% for the unmodified CNF aerogel to 44.2% at 1.5 wt % MEL–PA, together with greatly reduced weight loss and improved shape integrity after burning. Mechanistically, melamine released ammonia and water to dilute combustible gases, while phytic acid generated phosphorus-containing species that promoted dehydration and char formation, leading to efficient condensed-phase and gas-phase protection. This example highlights that interfacial assembly of P–N-rich structures on cellulose can provide flame-retardant effects comparable to graft-based systems, while retaining low thermal conductivity and the lightweight porous nature of the aerogel.

A further example is shown in Figure 5d,^107^ where a one-pot supramolecular assembly strategy was used to integrate phytic acid and polyethylenimine onto cellulosic microfibers, yielding flame-resistant insulating foams with a surface-confined P–N complex. Although this system is not a classical chain-growth graft copolymerization process, it similarly creates a strongly bound interfacial flame-retardant architecture on cellulose through pH-triggered assembly, with PEI acting as an anchoring layer between cellulose and phytic acid. The resulting foam exhibited a pronounced improvement in fire resistance, with the LOI increasing from 16.5% for the unmodified foam to 40.8%, together with clear self-extinguishing behavior after direct flame exposure. Mechanistically, phytic acid catalyzed dehydration and char formation, while PEI contributed nitrogen-containing species that promoted intumescence and suppressed flame propagation. SEM observations of the char layer further revealed that the original fibrous morphology was largely retained and fused into a compact honeycomb-like carbonaceous barrier, indicating efficient condensed-phase protection. This example highlights that interfacial assembly of P–N-rich macromolecular complexes on cellulose can provide graft-like durability and highly effective flame resistance, while still maintaining good thermal insulation performance.

Graft copolymerization offers excellent flexibility for introducing multifunctional flame-retardant polymers and enables precise control over the chemical structure of the grafted layer. The resulting materials generally exhibit superior durability compared to physically blended systems. However, grafting reactions often involve multiple processing steps, relatively high cost, and challenges associated with controlling the grafting density and reaction efficiency, which may limit large-scale industrial implementation.

### Challenges and Future Perspectives

Despite the substantial progress made in recent years, several challenges still limit the broader development and application of flame-retardant cellulose-based materials. These challenges involve not only flame-retardant efficiency itself but also durability, processability, scalability, and sustainability.

One major challenge is how to improve flame retardancy without sacrificing the intrinsic advantages of the cellulose. Many effective strategies, especially those involving intensive chemical modification or high additive loading, may compromise the mechanical strength, flexibility, moisture resistance, or thermal insulation performance. Achieving an optimal balance between fire safety and the inherent properties of cellulose, therefore, remains a key objective for future research.

Another important issue is the long-term stability under practical service conditions. Although many biobased and halogen-free systems have shown promising results, their resistance to moisture, repeated heating, mechanical deformation, and long-term aging is still insufficiently understood. More systematic durability tests, together with leaching and environmental assessments, are needed to evaluate their real application potential. Another challenge lies in balancing the multifunctionality and performance trade-offs. Although multifunctionality has become an important goal in the development of cellulose-based flame-retardant materials, the simultaneous optimization of flame retardancy, mechanical performance, thermal insulation, and environmental compatibility remains challenging. In many cases, enhancing one property inevitably affects the others. For example, high loadings of inorganic fillers or phosphorus-containing additives can increase the LOI, reduce the pHRR, and promote char formation, but excessive incorporation may disrupt the cellulose network, reduce flexibility, block pores, or weaken mechanical integrity. Similarly, dense coating layers or highly cross-linked structures can improve flame resistance and mechanical robustness, yet they may compromise porosity, increase thermal conductivity, and reduce thermal insulation performance. These trade-offs are particularly important for aerogels, foams, films, and textiles, where lightweight structures, flexibility, and thermal management are closely linked to practical application. Therefore, future studies should move beyond maximizing individual flame-retardant metrics and instead emphasize a balanced material design. Hierarchical porous structures, interfacial engineering, and synergistic flame-retardant systems may provide effective routes to reconcile flame retardancy with mechanical durability, insulation performance, and environmental sustainability.

In addition, the flame-retardant mechanisms of cellulose-based materials are often more complex than those currently described. Condensed-phase charring, gas-phase inhibition, and barrier effects usually occur simultaneously, but their relative contributions vary greatly, depending on the material system. Advanced in situ characterization and more precise mechanistic analysis will, therefore, be essential for moving from empirical formulation toward rational design.

Beyond conventional trial-and-error approaches, data-driven materials design and artificial intelligence (AI) are expected to accelerate the development of next-generation flame-retardant cellulose materials. Machine learning models integrated with high-throughput experimental data may enable rapid prediction of flame-retardant performance, thermal stability, and structure–property relationships. Such approaches could significantly reduce development time and facilitate the rational design of multifunctional systems with optimized fire safety, mechanical performance, and sustainability.

Another emerging direction involves the development of smart flame-retardant systems capable of responding to external stimuli, such as heat, smoke, humidity, or mechanical damage. Triggered char formation, self-healing coatings, and adaptive barrier structures may provide enhanced fire protection while maintaining material functionality during service. Such intelligent systems could open new opportunities for advanced building materials, wearable electronics, and transportation applications.

Finally, scalability is a practical bottleneck. Many high-performance systems are still developed through complex or energy-intensive laboratory processes, which limits industrial translation. Future efforts should place greater emphasis on simple, cost-effective, and scalable fabrication routes that are compatible with existing cellulose processing technologies.

Overall, the future of flame-retardant cellulose-based materials lies in combining fire safety with durability, multifunctionality, and sustainability. With continued progress in molecular design, structural engineering, and scalable processing, cellulose-based materials are expected to play an increasingly important role in next-generation fire-safe materials.

## Conclusions

Cellulose-based flame-retardant materials have attracted increasing attention as sustainable alternatives to conventional fire-safe materials because of their renewability, low density, biodegradability, and structural versatility. Recent advances have shown that effective flame retardancy can be achieved through multiple strategies, including physical blending, coating, chemical modification, and grafting copolymerization. These approaches regulate combustion behavior through different mechanisms such as promoting char formation, suppressing volatile release, creating physical barriers, and diluting combustible gases.

Among them, physical blending and coating offer relatively simple and scalable routes, whereas chemical modification and grafting copolymerization provide improved durability through stronger interfacial or covalent interactions. At the same time, increasing attention has been paid to biobased, halogen-free, and multifunctional systems that combine flame retardancy with desirable properties such as mechanical robustness, thermal insulation, and environmental compatibility. These developments highlight the importance of integrating molecular design with structural regulation across multiple length-scales.

Nevertheless, challenges remain in balancing fire safety with durability, processability, and sustainability. Future progress will depend on a deeper understanding of flame-retardant mechanisms, more reliable evaluation under realistic conditions, and the development of scalable fabrication strategies. Overall, with continued advances in materials design and processing, cellulose-based flame-retardant materials are expected to play an increasingly important role in the development of sustainable and high-performance fire-safe materials.
